# Curcumin-Based β-Diketo Ligands for Ga^3+^: Thermodynamic Investigation of Potential Metal-Based Drugs

**DOI:** 10.3390/ph15070854

**Published:** 2022-07-12

**Authors:** Matteo Mari, Debora Carrozza, Gianluca Malavasi, Ettore Venturi, Giulia Avino, Pier Cesare Capponi, Michele Iori, Sara Rubagotti, Silvia Belluti, Mattia Asti, Erika Ferrari

**Affiliations:** 1 Department of Chemical and Geological Sciences, University of Modena and Reggio Emilia, Via G. Campi 103, 41125 Modena, Italy; matteo.mari@unimore.it (M.M.); debora.carrozza@unimore.it (D.C.); gianluca.malavasi@unimore.it (G.M.); ettore-venturi@alice.it (E.V.); 2 Department of Chemical and Pharmaceutical Sciences, University of Trieste, Via L. Giorgieri 1, 34127 Trieste, Italy; gavino@units.it; 3 Radiopharmaceutical Chemistry Section, Nuclear Medicine Unit, Azienda USL-IRCCS Reggio Emilia, Via Amendola 2, 42122 Reggio Emilia, Italy; piercesare.capponi@ausl.re.it (P.C.C.); michele.iori@ausl.re.it (M.I.); sara.rubagotti@ausl.re.it (S.R.); mattia.asti@ausl.re.it (M.A.); 4 Department of Life Sciences, University of Modena and Reggio Emilia, Via G. Campi 183, 41125 Modena, Italy; silvia.belluti@unimore.it

**Keywords:** curcumin, keto–enol equilibrium, β-diketo ligands, gallium(III)-chelating agents, metal-based drugs

## Abstract

Curcumin is known for its therapeutic properties; among these, antioxidant, anti-inflammatory and anti-cancer ones stand out. Besides, curcumin metal complexes have shown widespread application in medicine and can be exploited as lead structures for developing metal-based drugs. Unfortunately, curcumin is poorly bioavailable, mainly due to its instability in physiological conditions; this weakness is tightly connected to the presence of the β-diketo moiety undergoing tautomeric equilibrium. Stability and metal-chelating ability can be tuned by modulating the electronic effects and steric hindrance close to the β-diketo moiety; in addition, formation of a metal complex shifts the tautomeric equilibrium towards the β-keto–enol form and increases stability in biological media. Among the metals used in clinical therapy, gallium nitrate has shown to have significant antitumor activity against non-Hodgkin lymphoma and bladder cancer, thus indicating that gallium-based drugs have potential for further development as antineoplastic agents with improved therapeutic activity. Curcuminoids have demonstrated high affinity for gallium(III), allowing the formation of stable positively charged M:L 1:2 β-diketonate complexes that benefit from the therapeutic activity of both the metal and the ligand. Seven new curcumin derivatives were synthesized and completely characterized. The new derivatives retain the solvent-dependent keto–enol tautomerism, with the prevalence of the diketo form in aqueous solution. Enhanced stability in simulated physiological conditions was observed in comparison to the lead compound curcumin. The presence of Ga^3+^ anticipates the dissociation of the enolic proton, allowing chelate complex formation, and simultaneously it shifts the tautomeric equilibrium towards the keto–enol form. A complete ^1^H/^13^C NMR and UV–Vis study was performed to define the metal-to-ligand stoichiometry ratio and the overall stability constants. In addition, we demonstrated that some of the derivatives have increased antiproliferative activity on colon cancer cells compared to curcumin and antioxidant properties. On the whole, the synthesized curcumin-based molecules may act as new gallium(III) chelators with improved stability with respect to curcumin and could open interesting perspectives for the development of novel therapeutic agents for cancer.

## 1. Introduction

Curcumin (CUR) is known for its therapeutic properties; among these, antioxidant, anti-inflammatory and anti-cancer ones stand out [1,2]. Unfortunately, curcumin is poorly water-soluble and bioavailable [3]; these are the drawbacks that hamper its great therapeutic properties and hold back its use in biomedical applications. This limit could be overcome by designing new methods of administration or synthesizing novel daughter compounds with modified chemical structures. The first strategy allows stabilizing the molecule and increases its bioavailability by reducing its metabolism and increasing the retention time in the bloodstream; recently, many studies have reported on the encapsulation of CUR into nanocarriers (i.e., liposomes, engineered nanoparticles or exosomes, EXOs) with promising preclinical results [4] even for neurological pathologies [5].

The second approach is based on a sort of SWOT analysis that points out particularly the weaknesses in the molecular structure related to the high instability in physiological media and the molecular moieties responsible for therapeutic activities. As the backside of the coin, huge efforts may easily account for failure or terrific success, since in silico investigations have the limit of theoretical models. To date, many SAR (structure–activity–relationship) studies have been performed to design different families of curcuminoids, as lately reviewed by Noureddin et al. [6]. Accordingly, the keto–enol structure is tentatively the main one responsible for fast CUR degradation in physiological conditions and poor bioavailability; this weakness is tightly connected to the presence of the β-diketo moiety undergoing tautomeric equilibrium, although the two tautomeric forms may also account for different biological properties [7].

Due to its C2V symmetry, CUR is characterized by only two tautomers: the keto–enol one (the average of the two equivalent keto–enol forms) and the diketo one. A more intricate situation is instead observed for asymmetric curcuminoids in which the two keto–enol tautomers are not equivalent and are entangled in complex equilibria [8,9]. The keto–enol tautomerism of CUR was investigated in depth, pointing out the prevalence of the keto–enol form in the solid state [10] and in apolar aprotic solvents, while the diketo form is more favored in polar/protic media, particularly in water [11]. Investigations of CUR in aqueous media are further complicated by its extremely low water solubility and by the fact that if spectroscopic techniques are employed, it is challenging to discriminate between the diketo form and the keto–enol form. In fact, mother CUR solutions are commonly prepared in a suitable organic solvent, typically methanol, ethanol or DMSO, that provides a solvation sphere around CUR enabling its dissolution in water but the organic solvation layer stabilizes the keto–enol form despite the polar/protic surrounding environment. Additionally, the structural modification of the keto–enol group by the insertion of a branching arm in the middle of it affects strongly the tautomeric equilibrium; typically, the equilibrium is shifted in favor of the diketo form as the bulkiness of the substituent is increased. In addition to thermodynamic features, recently, it was observed that the rate for reaching the tautomeric equilibrium is highly temperature-dependent, ranging from 700 s at 294 K to 200 s at 314 K [12]. 

CUR behaves as a weak triprotic acid due to the presence of the keto–enol moiety and the two phenol groups [13], hence, in a solution, proton dissociation equilibria occur. Particularly, the β-keto–enol moiety, due to its weak acid character and the presence of two oxygen atoms, can bind hard metal ions through the formation of a six-membered chelate ring. CUR has been demonstrated to form thermodynamically stable complexes with copper(II) [14,15,16], iron(III) and gallium(III), and other hard Lewis acids, as summarized by Wanninger et al. [13]. Formation of CUR metal complexes shifts the tautomeric equilibrium towards the β-keto–enol form and increases stability in biological media [14]. Furthermore, the stability in physiological conditions and the metal-chelating ability can be tuned by modulating the electronic effects and steric hindrance close to the β-diketo moiety. 

Besides, CUR metal complexes have shown widespread application in medicine [13,17] and can be exploited as lead structures for developing metal-based drugs. Among the metals used in clinical therapy, gallium nitrate has been shown to have significant antitumor activity against non-Hodgkin lymphoma and bladder cancer [18], thus indicating that gallium-based drugs have potential for further development as antineoplastic agents with improved therapeutic activity [19,20,21,22,23,24]. Curcuminoids demonstrated high affinity for gallium(III), allowing the formation of stable positively charged M:L 1:2 β-diketonate complexes that benefit from the therapeutic activities of both the metal and the ligand [25].

**Figure 1:**
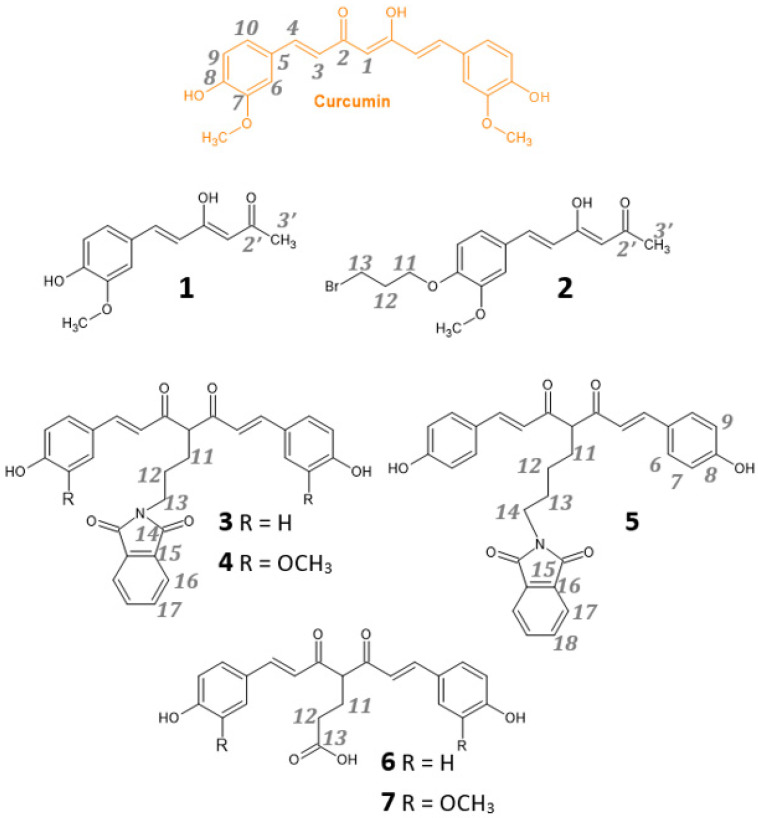
Chemical structures of curcuminoids and atom numbering for NMR signals’ assignment.

In this landscape, seven new curcumin derivatives were synthesized (Figure 1) and completely characterized. Two of them, compounds **1** and **2**, are asymmetric low-molecular-weight hemi-curcuminoids. 

The addition of a substituent in the center of the keto–enol moiety strongly affects the tautomeric equilibrium as well as acidity, stability and polarity, features woven with bioavailability and the related biological properties that can be tuned accordingly. Compounds **3**, **4** and **5** are symmetric substituted curcuminoids with an alkyl chain ending with a phthalimide group attached in the center of the keto–enol group. Phthalimide derivatives have recently shown similar activity to CUR in the inhibition of prostate cancer (PC) cell growth and migration, as well as in the modulation of the key molecular pathways involved in tumor progression and survival, opening the opportunity to develop new therapeutic drugs in aggressive PC tumors [26].

Finally, the carboxylic group in compounds **6** and **7** increases the solubility in aqueous media and provides an additional metal-binding group, as previously observed for K2A series [27].

The keto–enol tautomerism is hereafter investigated for all the new derivatives by means of UV–Vis and NMR spectroscopy, and stability in simulated physiological conditions in comparison to the lead compound CUR is estimated as well. The different ligands were compared for their gallium(III)-chelating ability to suggest a potential candidate for further in vitro investigations.

## 2. Results and Discussion

### 2.1. Synthesis

Syntheses of all the CUR derivatives were performed using a modified procedure of the Pabon reaction [28]. Differently from the condition applied according to Pabon, DMF was used as a solvent instead of ethyl acetate because the higher reaction temperature, 120 °C vs. 80 °C, increases the reactants’ and products’ solubility. The use of DMF increases the reaction yield and facilitates the work-up. 

The insertion of the chain in the keto–enol moiety was directly carried out in acetylacetone, the methylene group was activated in slightly basic conditions allowing the nucleophilic substitution to the Br derivative. All the curcuminoids were obtained with an average yield of around 50% except for compounds **2** and **5** that were obtained with 35% yield. 

### 2.2. Stability in Physiological Conditions

**Figure 2:**
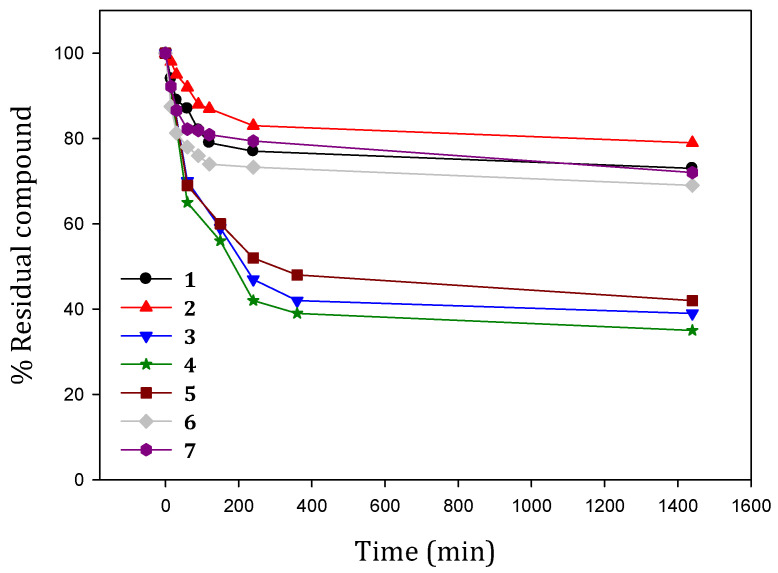
Stability profiles of the curcumin derivatives in simulated physiological conditions (PBS, 0.01 M; NaCl, 0.1 M; pH = 7.4; 37 °C in darkness). Residual percentage was calculated as A_t_ × 100/A_0_, where A_t_ and A_0_ stand for absorbance at λ_max_ at time *t* and time zero, respectively.

All the investigated compounds showed greater stability than the lead compound curcumin in physiological conditions [29], although ligands **1**, **2**, **6** and **7** are more stable than **3**, **4** and **5** (Figure 2). The reduction in conjugation for **1** and **2** and particularly the protection of the phenol group provides an improvement in stability of the physiological condition, a pillar feature for clinical applications. Carboxylic ligands **6** and **7** are found in their mono-dissociated negatively charged form at pH 7.4, able to form strong interactions with water molecules that probably prevent degradation equilibria. Considering the residual compound’s percentage after 4 h, the order of stability is as follows:

**2** > **7** > **1** > **6** > **5** > **3** > **4**

### 2.3. Tautomeric Equilibria in a Solution and Acid–Base Behavior of Ligands

Nuclear magnetic resonance provides valuable information on keto–enol tautomerism, although the main issue of this technique is the required concentration that should be around the mM range to gain quantitative data. All the investigated curcuminoids showed poor water solubility; hence, NMR analysis was performed in MeOD-*d*_4_ or DMSO-*d*_6_ solutions. 

**Figure 3:**
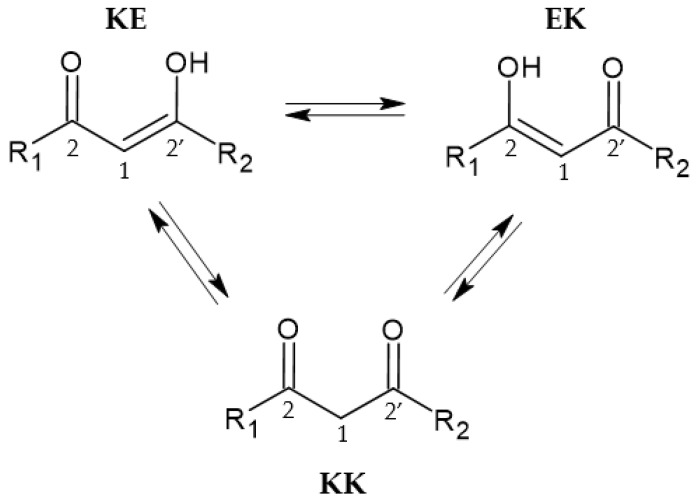
Schematic representation of tautomeric equilibria.

Bearing in mind the chemical structure of curcuminoids, two keto–enol forms (KE and EK) are in equilibrium with each other and with the diketo form (KK), as reported in Figure 3. Although the equilibrium between the two keto–enol forms (KE and EK) is too fast to be detected by NMR, in symmetrical structures (R_1_ = R_2_), such as curcumin, the two forms KE and EK are equivalent, and it is not possible to distinguish one from the other. In fact, the populations of the two tautomers (KE and EK) are equal, so the ^13^C chemical shift of C-2/C-2′ (~180 ppm) is an average of the carbonyl (~190 ppm) and enol (~170 ppm) carbons. For asymmetrical derivatives (R_1_ ≠ R_2,_ R_2_ = CH_3_, i.e., compounds **1** and **2**, Figure 1), the KE and EK forms are not equally populated and the equilibrium shifts strongly in favor of the EK form. ^13^C chemical shits of carbon 2 and 2′ are still an average of the chemical shifts of the two forms (KE and EK) in fast exchange with respect to the NMR timescale. However, in this situation, since the EK form is the prevailing species, the chemical shift of C-2 will be close to that of an enolic carbon while the chemical shift of C-2′ has a value typical of a carbonyl group. This hypothesis is verified by ^1^H,^13^C HMBC NMR experiments; in fact, the terminal methyl protons show long-range coupling with carbon C-2′ (~197 ppm) but not with C-2 (~178 ppm).

In a solution, with respect to the diketo moiety, the keto–enolic forms are usually stabilized by a strong intramolecular hydrogen bond (∆E, ~4 kcal/mol), this energy barrier diminishes as long as the *s-trans* diketo form interacts with a protic environment as an H-bond acceptor. In MeOD-*d*_4_ and DMSO-*d*_6_, the predominant form for CUR is the keto–enol one [30]; differently, all the new derivatives in methanol show the presence of both the KE and KK forms, with a high prevalence of the KE form according to the degree of π-conjugation and the bulkiness of the substituent in the middle of the keto–enol group, as summarized in Table 1. The addition of a bulky substituent within the keto–enol moiety accounts for an increase in the KK species, especially for compounds **6** and **7** that are mainly in the KK form.

**Figure 4:**
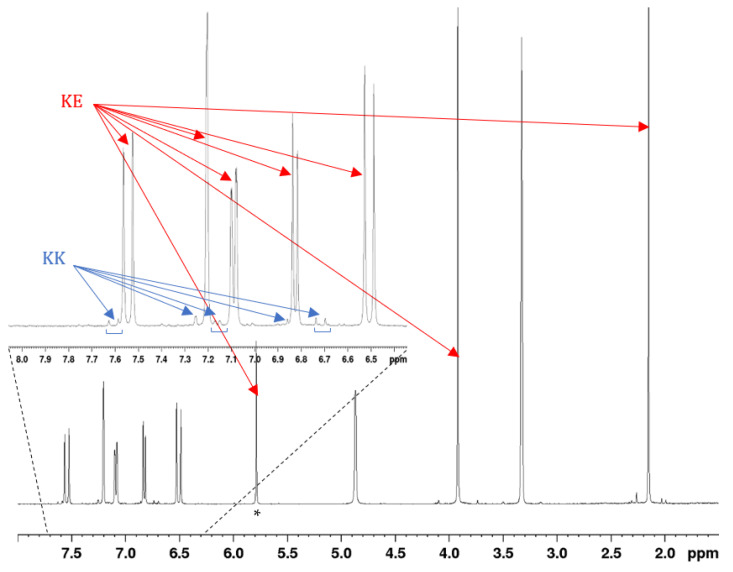
^1^H NMR spectrum of compound **1** in MeOD-*d*_4_ at 298 K (at 600 MHz). Blue and red arrows highlight the KK end KE tautomeric forms, respectively. * Enol vinyl signal.

The most informative NMR signal to investigate the keto–enol tautomerism should be the enol vinyl one, as reported in Figure 4 for compound 1. However, for highly conjugated structures such as curcuminoids, this proton undergoes fast exchange with deuterium of MeOD-*d*_4_; as a consequence, a disappearance of the enol vinyl proton is observed after little time from the sample preparation. It is possible to detect the presence of the tautomers through the protons on the double bond that are strongly affected by the tautomeric equilibrium. The integrated areas of this spin system were used to estimate the relative percentage of the tautomeric forms as reported in Table 1 and described in Experimental Section 3.5. 

For asymmetrical curcumin derivatives, in addition to the enol equilibria, it is also possible to observe in a solution the equilibrium between two structural isomers with respect to the double bond: E and Z isomers [31]. The energy gap between the E and Z forms is quite small (4/6 kcal/mol), and isomerization is possible. Although the E/Z equilibrium is shifted in favor of the E isomer, these two isomeric forms can easily be discriminated by ^1^H NMR on the basis of the scalar coupling constant ^3^J_HH_ between the two vinyl protons: 16 Hz for the E isomer and 12 Hz for the Z one. Since the two structural isomers exchange slowly in comparison to the NMR timescale, it is possible to observe two separate species corresponding to the E and Z forms, not an average of the two. 

UV–Vis spectroscopy can be particularly suitable to overcome NMR limitations caused by poor water solubility of CUR. In fact, curcuminoids, due to their extremely high molar extinction coefficient (ε_0_), can be analyzed in aqueous media (methanol <2%) despite their low solubility. CUR being a weak triprotic acid characterized by a keto–enol group, its spectroscopic properties are particularly dependent on the solvent and the pH conditions [32]. The enolic form is predominant in most of the solvents if CUR powder is dissolved in alcohol or DMSO before dilution in water. This species is the main one responsible for strong absorption with the maximum wavelength (λ_max_) ranging from 410 to 430 nm and weak absorption between 260 and 280 nm due to π→π* transitions [33]. Additionally, CUR presents a weaker transition n-π* (at ca. 375 nm) due to the KK form that increases when CUR is directly suspended in water. Keto–enol tautomerism is strongly affected by the presence of water molecules due to the different hydrogen bond interactions the keto and enol group can form as pointed out by Manolova et al. [8]. Compounds **1** and **2** show an intense absorption peak at 360 nm corresponding to the KE form; the blue shift compared to CUR is due to the reduced π-π conjugation. Derivatives **3**–**5** are characterized by a mixture of the KE (350–370 nm) and KK (420–450 nm) forms while **6** and **7** are mostly found in their KK form (330–350 nm) (Figure S1).

**Figure 5:**
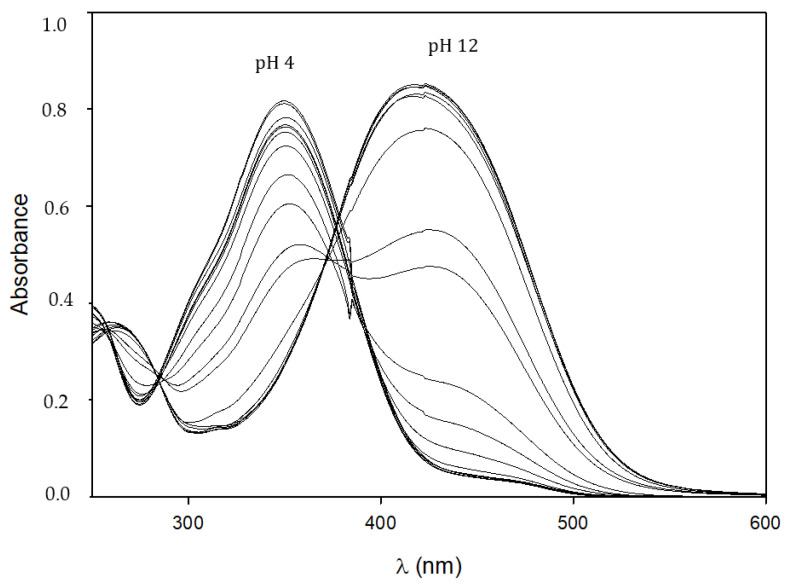
UV–Vis spectra of **7** at 25 °C in an aqueous solution (L = 50 μM) with increasing pH.

Spectrophotometric pH titrations were performed to evaluate the pH effect on the ligands for all the investigated compounds. According to the number of acid sites it is possible to group the ligands as HL (**2**), H_2_L (**1**), H_3_L (**3**,**4**,**5**) and H_4_L (**6**,**7**). As long as the pH is increased, a new absorption band is observed in the 450–500 nm range, as shown in Figure 5 for **7**, due to the dissociated form. 

Many equilibria occur as pointed out by the woven structure close to the isosbestic point, particularly dissociation reactions. Elaboration of the data allows estimating the overall protonation constants (logβ_MLH_) and pK_a_ values for all the compounds, as reported in Table 2. 

**Figure 6:**
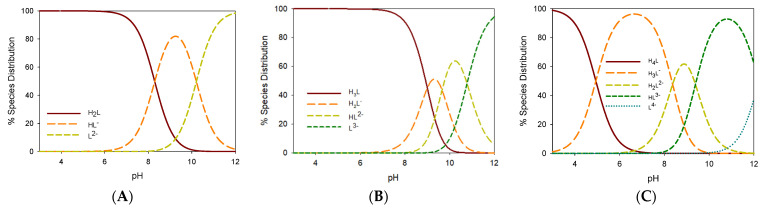
Species distribution curves for **1** (panel **A**), **4** (panel **B**) and **7** (panel **C**) at 25 °C ([L]_total_ = 10^−4^ mol L^−1^). The data were calculated on the basis of the *logβ_MLH_* values reported in Table 2.

Spectrophotometric titrations confirm, as recently suggested [29], that the phenol group in *para* position to the aliphatic chain is more acidic than the keto–enol moiety. Compound **2** is a monoprotic weak acid with a pK_a_ value of 10.903; likewise, compound **1** shows a similar value for the second dissociation (pK_a2_) that can only be attributed to the keto–enol group. For all the compounds, the proton dissociation of the first phenol group responsible for the main UV–Vis absorption around 450 nm, occurs within the pK_a_ range of 8.3–9.1. The pK_a_ values for keto–enol group are all in the range of 9.4–10.9 (Table 2), consistently with the previously reported keto–enol derivatives [29,35]. The second phenol group dissociates at extremely high pH; in fact, pK_a_ values range from 10.4 to 12.2. Compounds **6** and **7** have an additional acid group, the carboxylic one, that undergoes proton dissociation with a pK_a_ value close to 5. The species distribution curves for **1**, **4** and **7** are reported in Figure 6. All the compounds are mainly in their neutral form at physiological pH (7.4), except for **6** and **7** which are negatively charged as carboxylate anions. 

### 2.4. Gallium Complexing Ability

**Figure 7:**
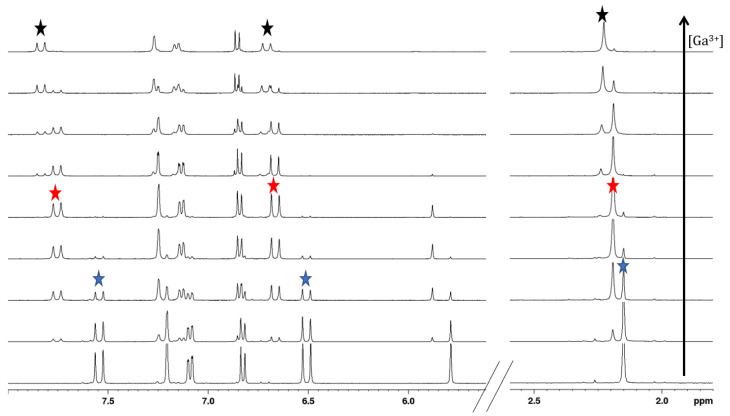
^1^H NMR spectra of compound **1** in MeOD-*d*_4_ at 298 K (at 600 MHz) at increasing addition of a Ga^3+^ MeOD-*d*_4_ solution up to the metal-to-ligand 1:1.5 molar ratio. The stars highlight resonances of protons H-1, H-3 and H-4 in the free ligand (blue), M:L 1:2 complex (red) and M:L 1:1 complex (black).

NMR can shed light on the metal–ligand interactions; particularly, these data allow not only defining the binding site and the metal-to-ligand molar ratio, but also investigating the actual equilibria in a solution, given that they are compatible with the NMR timescale. For all the free ligands, the enol proton of the keto–enol form was confirmed by the presence of a broad signal at 15–17 ppm (DMSO-*d_6_*/MeOD-*d_4_*) that disappears when the metal ion (Ga^3+^) is added to the solution. Apparently, this could be a proof of the proton dissociation of the ligand; indeed, the enol proton is extremely mobile and exchanges with the deuterated solvent after little time from sample preparation even in the absence of the metal ion. Hence, these data cannot be used as a solid proof of proton dissociation. Indeed, NMR titrations with Ga^3+^ quite clearly support the removal of the proton in favor of a trivalent metal ion; in fact, a downfield shift is observed for the protons close to the keto–enol moiety. However, for all the examined curcuminoids, the addition of Ga^3+^ at acidic pH (~5) instantly turns into the formation of a new set of downfield-shifted signals in a slow chemical exchange with respect to the NMR timescale. The high affinity of the keto–enol moiety for hard Lewis acid gallium(III) triggers an anticipated proton dissociation that enables the formation of an M:L 1:2 complex of the KE tautomer, the only one capable of binding the metal ion, as confirmed by the downfield shift of both allyl protons (H-3 and H-4, Figure 2) and ^13^C chemical shifts of the keto–enol group. The keto–enol group is an *O*,*O* bidentate chelator that allows the formation of a highly stable six-membered ring with the metal ion. The reaction is fast, and the formation of the metal complex is observed immediately after the addition of the metal ion. As long as the KE form reacts with the metal, the tautomeric equilibrium is shifted towards the depletion of the KK form in favor of the KE one. The complete disappearance of KK is observed within 24 h for all the ligands. An increase in Ga^3+^ concentration till the 1:1 M:L molar ratio allows the formation of a complex species with metal-to-ligand molar ratio 1:1 as suggested by new downfield-shifted signals and the concurrent disappearance of the 1:2 M:L complex. Figure 7 reports the titration with Ga^3+^ in MeOD-*d*_4_ for compound **1** (compound **7** is reported in Figure S2). 

**Figure 8:**
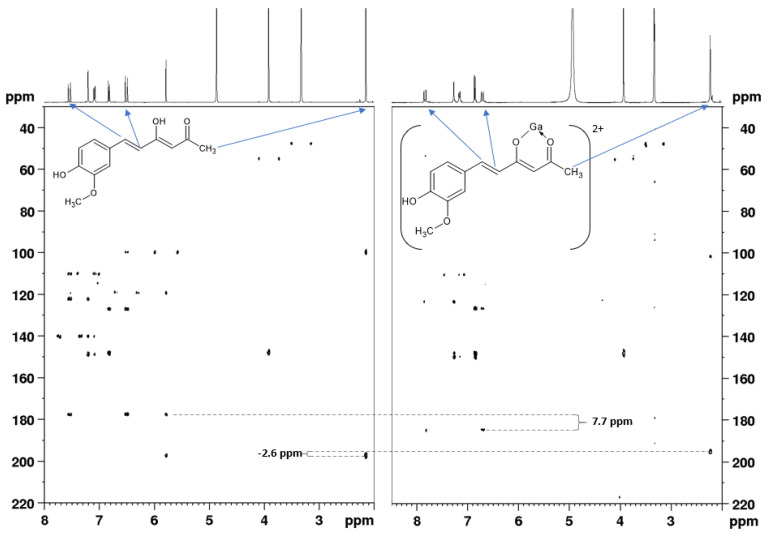
^1^H,^13^C HMBC NMR spectra in MeOD-*d*_4_ at 298 K (at 600 MHz) of compound **1** (**left**) and its gallium(III) complex with the metal-to-ligand 1:1 molar ratio (**right**).

The downfield shifts for protons H-3 and H-4 in the M:L 1:1 complex range between 0.15 and 0.3 ppm (90–180 Hz at 600 MHz) for all ligands, C-3 and C-4 are downfield-shifted (around 3–4 ppm). The shift observed for C-2 is close to 1 ppm for all the compounds except for **1** and **2**. In these compounds, carbons C-2 and C-2′ are not chemical shift-equivalent since only one of the two keto–enol forms is observed in the solution, the EK form, in which the keto group is in the α position to the methyl one, as confirmed by ^1^H,^13^C HMBC spectra (Figure 8—left). The EK tautomer is favored by higher π–π conjugation that is gained when the enol group is directly attached to the vinyl chain. When Ga^3+^ is bound to the keto–enol group (Figure 8—right), C-2 is upfield-shifted by the exchange of the proton with the metal ion, ∆δ(^13^C) = 6.9 ppm from 177.8 to 184.7 ppm and ∆δ(^13^C) = 7.7 from 177.0 to 184.7 ppm for compounds **1** and **2**, respectively. C-2′ is less affected by the insertion of the metal ion, and it results in downfield shifting, ∆δ(^13^C) = −2.6 ppm from 197.9 to 195.3 ppm and ∆δ(^13^C) = −2.1 ppm from 197.9 to 195.8 ppm for **1** and **2**, respectively. 

Finally, NMR data suggest that the carboxylic group of **6** and **7** is probably not involved in metal coordination; in fact, we observed a higher downfield shift for the proton closer to the KE group (H-11) than the farther one (H-12), ∆δ(^1^H) = 24 Hz and 50 Hz vs. 11 Hz and 38 Hz for M:L 1:2 and M:L 1:1, respectively (Figure S2). The weak interaction of the carboxylic group with the metal ion could be explained by the absence of a beneficial chelate effect connected to the long length of the aliphatic chain itself.

**Figure 9:**
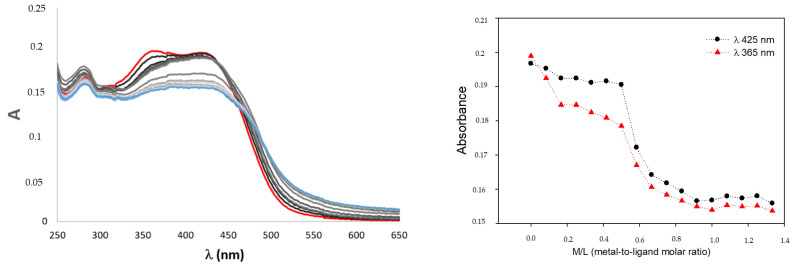
Spectrophotometric titration of **4** with a Ga^3+^ solution in acidic conditions (pH ~5) up until the M/L 1:1.3 is reached (**left**) and plots of absorbance at different λ_max_ vs. M/L (**right**) at 25 °C ([L] = 50 μM).

As reported above, curcuminoids have characteristic UV–Vis absorption spectra; hence, formation of metal complexes can be investigated using this technique. When Ga^3+^ is added to the ligand aqueous solution under acidic conditions (pH ~5), the absorbance decreases and a red shift of the KE maximum is observed, suggesting the formation of the metal complex (Figure 9). By plotting the absorbance vs. the metal-to-ligand molar ratio, the formation of M:L 1:2 and 1:1 complexes can be suggested, confirming the outcomes of NMR titrations. The so-formed metal complexes are extremely stable (residual compound’s percentage (8 h) >90% for all the ligands), suggesting that fixing the keto–enol moiety into the KE form in the formation of coordination bonds highly enhances the kinetic stability of the ligand itself.

In order to assess the metal-to-ligand molar ratio, LC/MS experiments were carried out supporting the formation of M:L 1:2 complexes for compounds **1**–**5** and of M:L 1:1 species for compounds **6** and **7** (Table S1).

A complete investigation of the thermodynamic stability of metal complexes in an aqueous medium was then carried out through pH-metric UV–Vis titrations. 

**Figure 10:**
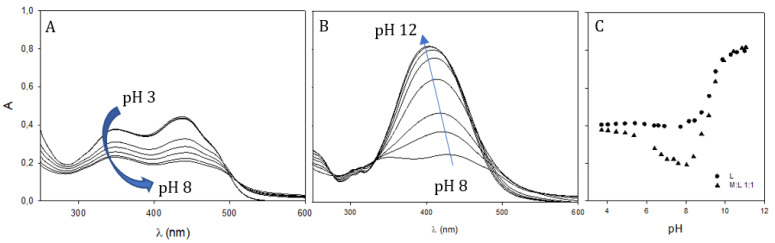
UV–Vis spectra of the Ga^3+^: **6** 1:1 molar ratio at 25 °C (L = 50 μM) on increasing pH. Panel (**A**)—pH range of 3–8; panel (**B**)—pH range of 8–12; panel (**C**)—plot of absorbance at 405 nm vs. pH for the free ligand (circles) and the metal-ligand system (triangles).

The increase in pH in M:L 1:1 systems causes an initial decrease in the intensity of the maximum band, followed by the appearance of a new intense absorption of deprotonated systems around 420–430 nm (Figure S3). The presence of the metal ion in the solution anticipates the dissociation of the keto–enol group of 3–4 pK_a_ units, as suggested by the plot of absorbance at λ_max_ vs. pH for compound **6** as an example (Figure 10). On increasing pH starting from extremely acidic conditions (pH 3), a decrease in absorbance at 405 nm is observed until pH reaches a value around 8 (Figure 10A). In this pH interval, the spectral profile remains unchanged, with the coexistence of both KE and KK forms suggesting that the conversion of the KK form into the KE form, due to metal chelation, is slow and probably hampered by the formation of a strong network of hydrogen bonds between keto groups and water molecules. When pH is raised above 8 (Figure 10B), the absorbance band of dissociated phenol appears at 420 nm and increases in intensity until pH 12. It is possible to observe a slight blue shift of λ_max_ within this pH range.

The stability constants of the complexes were calculated taking into account the possible complexes at the M:L 1:1, 1:2 and 1:3 molar ratios, but only for compounds **1** and **2** the refinements converged completely. For bulky ligands (**3**,**4**,**5**,**6**,**7**), it was necessary to exclude M:L 1:3 to reach convergence due to their steric hindrance that prevents the rearrangement of three ligands in the octahedral coordination sphere of Ga^3+^.

To compare ligands with different proticity, logK_11_, logK_12_ and logK_13_ were calculated according to the equations reported in the caption of Table 3.

**Figure 11:**
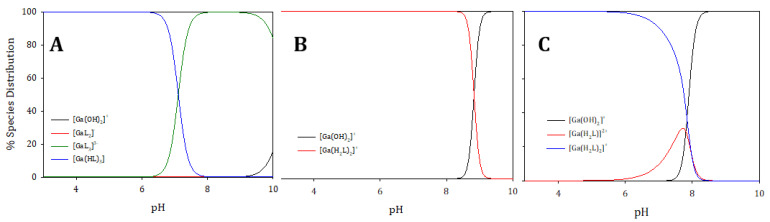
Species distribution curves for **1** (panel **A**), **4** (panel **B**) and **7** (panel **C**) at 25 °C (L_total_ = 10 μM; Ga^3+^_total_ = 1 μM). The data were calculated based on the logβ values reported in Table 3.

The species distribution curves for **1**, **4** and **7** are reported in Figure 11.

Compounds **1**–**5** have similar logK values, while ligands **6** and **7,** probably due to their steric hindrance, are unable to form metal complexes with a smaller M:L molar ratio and show lower K values. This decreased affinity for the metal ion is highlighted by the species distribution curves (Figure 11C); indeed, formation of Ga(III) hydroxide species is observed slightly above the physiological pH (>7.5).

To consistently compare the chelating abilities for Ga(III), *pGa* values (Table 3) were calculated as −log[Ga^3+^]_free_. The use of pM dates back to the paper of Harris et al. [37], in which it was used for the first time to express and relate the iron(III)-sequestering ability of siderophores. Among the investigated chelators, only the phthalimide derivatives (**3**, **4** and **5**) showed interesting *pGa* values, quite close to those of renowned gallium chelators such as DFOB (*pGa* = 21.6) [38] or the recently developed PrP9, a triazacyclononane-based phosphinate ligand (*pGa* = 23.1) [39], and the hydroxamate chelators lately reported by Toporivska et al. [40] (*pGa* 21.9 (min)–25.4 (max)). The difference of two orders of magnitude between compounds **3**/**4** and **5** reflects the steric hindrance provided by the phthalimide group. In fact, when this bulky moiety is separated from the keto–enol moiety by a longer spacer (butyl vs. propyl chain), metal chelation is more effective and logK_12_ is greatly increased (16.1 vs. 14.05/14.45), suggesting the formation of a more stable M:L 1:2 complex species.

Finally, the kinetic stability of metal complexes at the M:L 1:1 molar ratio were investigated by UV–Vis spectroscopy in physiological conditions (PBS pH 7.4). Formation of coordination bonds to the metal strongly stabilizes the ligand, and for all the metal complexes, the percentage of the residual compound was greater than 85% within 8 h (data not shown).

### 2.5. Transmetalation Reactions of Selected Curcumin Derivatives ***4*** and ***5***

Compounds **4** and **5** were selected to evaluate iron-chelating ability by spectrophotometric analysis in the same conditions as the ones applied to carry out investigations on Ga^3+^ systems. The calculated logβ_124(Fe)_ values were 56.2 (2) and 58.6 (4) for **4** and **5**, respectively. From the difference between the logβ values of the complexes with two metal ions, it is possible to calculate the apparent stability constant K (logK = logβ_124(Fe)_ − logβ_124(Ga)_) associated with the transmetalation reaction (Equation (1)).

(1) $${\mathrm{Fe}}^{3+}+[\mathrm{Ga}(H_{2}L{)}_{2}{]}^{+}\;⇆\;{\mathrm{Ca}}^{3+}+[\mathrm{Fe}(H_{2}L{)}_{2}{]}^{+}$$

LogK values are small for **4** and **5** (0.9(3) and 1.2(7), respectively), confirming a similar sequestering ability towards Ga^3+^ and Fe^3+^.

### 2.6. Antioxidant Activity and Cell Viability Assays

Compounds **4** and **5** are characterized by the presence of two phenolic groups that could be responsible for additional antioxidant properties, so the DPPH radical-scavenging assay was carried out for the free ligands **4** and **5** and their Ga^3+^ complexes (Figure S4). Both compounds showed similar IC_50_ values (20 μM and 32 μM for **4** and **5**, respectively) and close to that reported for curcumin (13 μM [14]).

**Figure 12:**
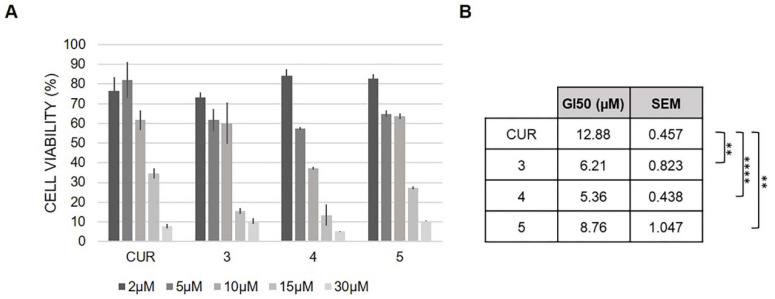
Biological activity of the selected curcumin derivatives on human colorectal HCT116 cancer cells. Panel (**A**): dose-dependent effect of curcumin derivatives administration for 48 h on the cell growth of HCT116 cancer cells as assessed by the MTT assay. Panel (**B**): GI_50_ values and statistical significance compared to curcumin are indicated as the means ± SEM (unpaired *t*-test: ** *p* < 0.01, **** *p* < 0.0001).

To establish the potential antitumor activity of compounds **3**, **4** and **5**, we evaluated their effects on the proliferation of HCT116 colon carcinoma cells, a cellular tumor model previously used for the characterization of curcumin derivatives [41]. We performed dose response experiments (Figure 12A) and calculated GI_50_ values as the concentration of molecules that induces 50% growth inhibition after 48 h of treatment (Figure 12B). Curcumin and compound **4** were used as reference drugs since we had previously reported their antiproliferative activity in HCT116 cells [26]. Comparing curcumin with the synthesized derivatives revealed that the conjugation of phthalimide significantly increases their antiproliferative activity in HCT116 cells (Figure 12). Besides, **5** showed a major efficacy in terms of growth inhibitory activity (GI_50_) compared to curcumin. 

## 3. Materials and Methods

### 3.1. General Procedures

All the chemicals and solvents were purchased with the highest purity grade available and used without further purification unless otherwise specified; pH measurements were carried out using a calibrated pH-meter (Mettler-Toledo). Liquid chromatography/mass spectrometry (LC/MS) was performed on an Agilent 6300 Ion Trap LC/MS System equipped with an electrospray ionization (ESI) interface. Elemental analysis was performed on a Thermo Scientific™ FLASH 2000 CHNS Analyzer.

All the reaction intermediates were purified as specified in the following procedures and their purity (≥95%) was checked by a combination of LC/MS, NMR and elemental analysis. 

Atom numbering of the NMR data refers to Figure 1. 

### 3.2. Synthesis

*4-(3-bromopropyl)-3-methoxybenzaldehyde*

1,3-dibromopropane (3 mL) is slowly added to a suspension of vanillin (1.5 g) and K_2_CO_3_ (1.9 g) in 15 mL of dimethylformamide (DMF). The mixture is kept under stirring at 75 °C overnight, then the temperature is cooled down, the solid is filtered off and the solution is neutralized by adding 10% CH_3_COOH aqueous solution. The product is extracted in ethyl acetate (EtOAc), the organic phases are then collected, washed with NaHCO_3_ and brine and finally dried over Na_2_SO_4_. After filtering off the salts, the solvent is removed under reduced pressure. The raw product is purified by flash column chromatography (silica, gradient (*v*/*v*) petroleum ether (EtPet): ethyl acetate (EtOAc) 100:0→70:30). Light yellow solid, 27% yield. Elemental analysis for C_11_H_13_O_3_Br (267.08 g/mol): calc. C, 50.19%; H, 5.27%; exp. C, 50.02%, H, 5.49%. LC/MS (ESI): *m/z* 287.0 [M + H]^+^. ^1^H NMR (δ (ppm), CDCl_3_): 9.87 (1H), 7.43 (d, 1H), 6.99 (d, 1H), 7.46 (dd, 1H), 4.16 (t, 2H), 2.10 (m, 2H), 3.53 (t, 2H), 3.94 (OCH_3_, s, 3H).

*(3Z,5E)-4-hydroxy-6-(3-methoxy-4-methylphenyl)hexa-3,5-dien-2-one (***1***)*

The procedure was adapted from Pabon’s CUR synthesis [28]; actually, boron acetylacetonate is obtained by reacting B_2_O_3_ (0.72 g) and 1.1 mL of acetylacetone (acac) in DMF (11 mL). The mixture is kept under reflux at 80 °C for 30 min, then 11 mL tributyl borate is added, and the mixture is kept under stirring at 80 °C overnight. After 16 h, the suspension turns orange–red and becomes dark red when vanillin is added (0.265 g). *n*-butylamine (145 µL in 0.5 mL of DMF) is slowly dropped over 1 h. The reaction batch is maintained under heating and stirring for 6 more hours, then the system is cooled down to r.t., acidified by adding 40 mL of 0.5 M HCl and kept under stirring for 1 h. The aqueous phase is extracted with EtOAc three times, the organic phases are collected, washed with NaHCO_3_, brine and eventually dried under Na_2_SO_4_. After filtering off the salts, the solvent is removed under reduced pressure. The raw product is purified by flash column chromatography (silica, gradient (*v*/*v*) EtPet: EtOAc 100:0→70:30). Yellow powder, 45% yield. Elemental analysis for C_13_H_14_O_4_ (234.25 g/mol): calc. C, 66.66%, H, 6.02%; exp. C, 66.52%, H, 6.18. LC/MS (ESI): *m/z* 235.1 [M + H]^+^. ^1^H NMR (δ (ppm), MeOD-*d*_4_): 7.55 (H-4, d, 1H), 7.20 (H-6, d, 1H), 7.09 (H-10, dd, 1H), 6.82 (H-9, dd, 1H), 6.51 (H-3, d, 1H), 5.79 (H-1, s, 1H), 3.92 (OCH3, s, 3H), 2.15 (H-3′, s, 3H).

*(3Z,5E)-6-[4-(3-bromopropoxy)-3-methoxyphenyl]-4-hydroxyhexa-3,5-dien-2-one (* **2** *)*

Compound **2** was obtained similarly to **1**, but 4-(3-bromopropyl)-3-methoxybenzaldehyde was used instead of vanillin. Yellow powder, 35% yield. Elemental analysis for C_16_H_19_BrO_4_ (355.22 g/mol): calc. C, 54.10%, H, 5.39%; exp. C, 53.89%, H, 5.48%. LC/MS (ESI): *m/z* 355.1 [M + H]^+^. ^1^H NMR (δ (ppm), MeOD-*d*_4_): 2.16 (H-3′, s, 3H), 5.81 (H-1, s, 1H), 6.56 (H-3, d, 1H), 7.56 (H-4, d, 1H), 7.25 (H-7, dd, 1H), 7.00 (H-9, dd, 1H), 7.17 (H-10, dd, 1H), 4.18 (H-11, t, 2H), 2.34 (H-12, m, 2H), 3.67 (H-13, t, 2H), 3.91 (OCH_3_, s, 3H).

*(2-((4Z,6E)-5-hydroxy-7-(4-hydroxyphenyl)-4-((E)-3-(4-hydroxy-3-ethoxyphenyl) acryloyl) hepta-4,6-dien-1-yl)isoindoline-1,3-dione (***3***)*

Compound **3** was synthesized as previously reported [21]. Orange powder, 40% yield. Elemental analysis for C_30_H_25_NO_6_: calc. C, 72.72%, H, 5.09%, N, 2.83%; exp. C, 73.06%, H, 5.21%, N, 2.75%. LC/MS-IT: *m/z* 496.6 (M + H)^+^. ^1^H NMR (DMSO-*d*_6_): 7.00 (H-3, d, 2H), 7.58 (H-4, d, 2H), 7.54 (H-6/H-10, d, 4H), 6.79 (H-7/H-9, d, 4H), 2.65 (H-11, t, 2H), 1.83 (H-12, m, 2H), 3.77 (H-13, t (broad), 2H), 7.88 (H-16, m, 2H), 7.85 (H-17, m, 2H).

*(2-((4Z,6E)-5-hydroxy-7-(3-methoxy-4-hydroxyphenyl)-4-((E)-3-(4-hydroxy-3-ethoxyphenyl) acryloyl) hepta-4,6-dien-1-yl)isoindoline-1,3-dione (***4***)*

Compound **4** was synthesized as previously reported [26]. Light-orange powder, 50% yield. Elemental analysis for C_32_H_29_NO_8_ (555.57 g/mol): calc. C, 69.18%, H, 5.26%, N, 2.52%; exp. C, 69.02%, H, 5.39%, N, 2.83%. LC/MS-IT: *m/z* 556.2 (M + H)^+^. ^1^H NMR (δ (ppm), DMSO-d_6_): 7.00 (H-3, d, 2H), 7.58 (H-4, d, 2H), 7.54 (H-6/H-10, d, 4H), 6.79 (H-7/H-9, d, 4H), 2.65 (H-11, t, 2H), 1.83 (H-12, m, 2H), 3.77 (H-13, t (broad), 2H), 7.88 (H-16, m, 2H), 7.85 (H-17, m, 2H).

*2-((4Z,6E)-5-hydroxy-7-(4-hydroxyphenyl)-4-((E)-3-(4-hydroxy-3-ethoxyphenyl) acryloyl) hepta-4,6-dien-1-yl)isoindoline-1,3-dione (***5***)*

Compound **5** was synthesized following a procedure previously reported [26]. 2,4-pentandione (25 mmol) is added to a suspension of K_2_CO_3_/KI (50/3 mmol) in dry acetone (15 mL) at 80 °C and kept under stirring for 1 h. Then, a solution of 2-(4-bromobutyl)-1H-isoindole-1,3(2H)-dione (25 mmol) in dry acetone (5 mL) is added dropwise and kept under magnetic stirring overnight at 80 °C. The solution is then cooled down to *r.t*., diluted with aqueous NH_4_Cl and extracted twice with DCM. The organic phases are washed with brine and dried over Na_2_SO_4_. After solvent removal under reduced pressure, a yellow sticky product is obtained that requires further purification by flash column chromatography. Orange powder, 34% yield. Elemental analysis for C_31_H_27_NO_6_ (509.55 g/mol): calc. C, 73.07%, H, 5.34%, N, 2.75%; exp. C, 73.25%, H, 5.44%, N, 2.85%. LC/MS-IT: *m/z* 510.2 (M + H)^+^. ^1^H NMR (δ (ppm), CDCl_3_): 7.72 (H-18, m, 2H), 7.81 (H-17, dd, 2H), 3.83 (H-14, m (broad), 2H), 1.92 (H-13, m, 2H), 1.62 (H-12, m, 2H), 2.67 (H-11, t, 2H), 6.98 (H-3, d, 2H), 7.73 (H-4, d, 2H), 7.22 (H-10, dd, 2H), 7.18 (H-9, d, 2H), 6.99 (H-6, d, 2H), 4.03 (OCH_3_, s, 6H).

*(6E)-7-(4-hydroxyphenyl)-4-[(2E)-3-(4-hydroxyphenyl)prop-2-enoyl]-5-oxohept-6-enoic acid (***6***)*

Compound **6** was synthesized following the procedure reported by Ferrari et al. starting from 4-acetyl-5-oxohexanoic acid obtained as previously reported [27]. Light orange powder, 47% yield. Elemental analysis for C_22_H_20_O_6_ (380.39 g/mol): calc. C, 69.46%, H, 5.30%; exp. C, 69.31%, H, 5.60%. LC/MS-IT: *m/z* 381.1 (M + H)^+^. ^1^H NMR (δ (ppm), MeOD-*d_4_*): 2.35 (H-12, t, 2H), 2.20 (H-11, m, 2H), 6.79 (H-3, d, 2H), 7.69 (H-4, d, 2H), 7.43–760 (H-6/10, m, 4H), 6.60–6.88 (H-7/9, m, 4H).

*(6E)-7-(3-methoxy-4-hydroxyphenyl)-4-[(2E)-3-(3-methoxy-4-hydroxyphenyl)prop-2-enoyl]-5-oxohept-6-enoic acid (***7***)*

Compound **7** was synthesized following the procedure reported by Ferrari et al. [22] starting from 4-acetyl-5-oxohexanoic acid obtained as previously reported [24]. Orange-red powder, 58% yield. Elemental analysis for C_24_H_24_O_8_ (440.44 g/mol): calc. C, 65.45%, H, 5.49%; exp. C, 65.26%, H, 5.74%. LC/MS-IT: *m/z* 441.2 (M + H)^+^. ^1^H NMR (δ (ppm), CDCl_3_): 7,72 (H-3, d, 2H), 6.82 (H-4, d, 2H), 7.25 (H-6, dd, 2H), 6.84 (H-9, dd, 2H), 7.16 (H-10, dd, 2H), 2.24 (H-11, t, 2H), 2.38 (H-12, t, 2H), 3.90 (OCH_3_, s, 6H). 

### 3.3. Kinetic Stability of Ligands in Physiological Conditions

The chemical stability of ligands and metal complexes at the M:L 1:L molar ratio at 37 °C in darkness was evaluated by UV−Vis spectroscopy as a change in absorbance in the 250−600 nm range over an overall period of 8 h. Solutions (5.0 × 10^–5^ mol L^–1^) of the ligands were prepared in 0.1 mol L^–1^ phosphate-buffered solution (PBS) at pH 7.4. Spectra were recorded every 30 min. All profiles were linearized as the hyperbolic function defined by Equation (2), which represents an empirical model that describes drug decomposition or release well [42]:

(2) $$\frac{t}{f_{\%}}=at+b$$

where *f*_%_ is the percentage of the residual compound at time *t* (min) referred to the starting concentration at time zero determined by reading the absorbance at λ_max_. 

### 3.4. UV–Visible Spectroscopy

UV–visible spectra were recorded with a JASCO V-570 UV/Vis/NIR spectrophotometer at 298 K in the 250–600 nm spectral range employing quartz cells (1 cm optical path). To perform spectrophotometric titrations, ligands (denoted generally as L in the following) were dissolved in methanol to give a mother solution (2.50 mM), that was then diluted in water in order to obtain a final concentration of 50 μM. The metal/ligand (M:L) solutions were obtained by adding appropriate quantities of Ga(NO_3_)_3_ as methanol solutions (5–10 mM) in order to obtain the metal-to-ligand 1:1 molar ratio (L = 50 μM and V = 25 mL). For both L and M:L systems, pH (initial value of about 5) was varied by adding small amounts (<1 μL) of concentrated NaOH (4 M) up to ca. pH 11, thus to consider volume variations negligible. To evaluate the complexation ratio, constant additions of the M solution (10 µL each time, M = 10^–2^ M) were dropped into the ligand solution (V = 25 mL, L = 50 μM) using a micropipette in order to reach different M:L molar ratios. A constant ionic strength (NaNO_3_, 0.1 M) was maintained in all experiments. Spectrophotometric investigations of iron(III) systems were performed in the same conditions; the Fe^3+^ solution was freshly prepared in methanol starting from Fe(NO_3_)_3_·9H_2_O. The overall protonation constants (logβ_qr_) and the overall stability constants of metal complexes (logβ_pqr_) were evaluated from spectrophotometric data. The stability constants (β_pqr_), defined by the following equations:

(3) $$pM^{m+}+qL^{l^{-}}+rH^{+}⇌M_{p}L_{q}H_{r}^{\left(mp-lq+r\right)}$$

(4) $$\beta_{pqr}=\frac{\left[M_{p}L_{q}H_{r}^{\left(mp-lq+r\right)}\right]}{[M^{m+}{]}^{p}\cdot [L^{l-}{]}^{q}\cdot {\left[H^{+}\right]}^{r}}$$

where *L* is the ligand in the completely dissociated form and *H* is proton, were refined using least-squares calculation in HypSpec [36]. The results of least-squares calculation include the standard deviations and correlation coefficients of the refined parameters. The quantities are obtained by performing error propagation calculation from the experimental errors onto the parameters. The stability constant refinement furnishes least-squares estimates of the standard deviation, σ, of the stability constant β. The error on logβ is calculated as follows: σ(logβ) = [log(β + σ) − log(β − σ)]/2.

Hydroxide species, such as [Ga(OH)_2_]^+^, were taken into account for calculations. The hydrolysis constants for Ga^3+^ are as follows: logβ_1,1,0_ = −3.93; logβ_2,1,0_ = −7.73; logβ_3,1,0_ = −12.38; logβ_4,1,0_ = −15.96; logβ_32,13,0_ = −66.3; logβ_3,1,0(s)_ = −3.54 [43]; pGa is defined as (−log[Ga^3+^]_free_) and is calculated at specific conditions (Ga^3+^_total_ = 1 μM, L_total_ = 10 μM, pH 7.4, 25 °C). For the calculation of the iron(III) overall stability constants, the presence of [Fe(OH)]^2+^, [Fe(OH)_2_]^+^ and [Fe(OH)_3_] species was taken into account [44].

### 3.5. Nuclear Magnetic Resonance

NMR spectra were recorded by means of a Bruker Biospin FT-NMR AVANCE III HD (600 MHz) spectrometer equipped with a CryoProbe BBO H&F 5 mm in inverse detection. The nominal frequencies were 150.90 MHz for ^13^C and 600.13 MHz for ^1^H, respectively. For each sample, 1–5 mg were weighed and diluted up to 0.6 mL with a proper solvent in a 5 mm NMR tube; 90° pulse was calibrated for each sample, and standard NMR parameters were used to achieve quantitative results (relaxation delay, 10 s). Proton and carbon chemical shifts are given in parts per million (ppm) versus external TMS and were determined by reference to the solvent residual signals. Typical 2D homo- and heteronuclear techniques were used for assignment, i.e., ^1^H,^1^H COSY, ^1^H,^13^C HSQC, ^1^H,^13^C HMBC. NMR investigations of Ga^3+^:L systems were performed in MeOD-*d*_4_ by adding small amounts of a Ga(NO_3_)_3_ solution (10 μL each) to 1 mM ligand solution (0.6 mL) until reaching the desired metal-to-ligand molar ratio. The complexation reaction is fast, and spectra were registered few minutes after the addition. Two-dimensional ^1^H,^13^C heteronuclear multiple bond correlation (HMBC) experiments (*hmbcgpndqf* in Bruker library) were performed via heteronuclear zero and double quantum coherence, optimized on long-range couplings (^3/4^J_HC_ = 8 Hz), with no decoupling during acquisition, and gradient pulses were used for selection. The relative percentages of the isomeric forms were estimated on the basis of the NMR data. Particularly, ^1^H NMR spectra were acquired in quantitative conditions being D1 (relaxation delay) at least five times the T1 of the slowest relaxing signal of interest. The raw data were Fourier-transformed, and then the processing was performed by zero filling (SI = TD), line broadening (lb 0.3 Hz), accurate phasing and correcting the baseline. The most isolated signals were selected for integration, i.e., the vinyl protons (H-3 and H-4), and an average of the integrated area (*A*) of H-3 and H-4 was used for relative quantitation, according to the following equation:

$$\%{Tautomer}_{i}=\frac{(A_{\left(H-3\right)i}+(A_{\left(H-4\right)i})/2}{\sum_{i}(A_{\left(H-3\right)i}+(A_{\left(H-4\right)i})/2}\times 100$$

### 3.6. Antioxidant Activity (DPPH Assay)

The antioxidant activity of compounds **4** and **5** and their Ga^3+^ metal complexes at the M:L 1:1 and 1:2 molar ratio was evaluated in terms of hydrogen-donating or radical scavenging ability using a stable DPPH radical, 1,1-diphenyl-2-picrylhydrazyl radical, following the procedure previously reported [26]. The percentage of inhibition (%In) of the DPPH radical by each sample was calculated according to the following formula:

$$\%In=\frac{A_{0}-A_{t}}{A_{0}}\times 100$$

where *A*_0_ represents the absorbance of the control (DPPH radical) at time 0, while *A_t_* refers to the absorbance of the mixture antioxidant/DPPH at time t (120 min). Values of absorbance were corrected considering volume dilution. All the determinations were performed in duplicate.

### 3.7. Cell Viability Assay

Human colorectal carcinoma HCT116 cells (#91091005, ECACC) were grown in an IMDM medium (Biowest) supplemented with 2 mM glutamine, 100 IU/mL penicillin, 100 μg/mL streptomycin and 10% FBS (Gibco). The cell lines were grown at 37 °C in a humidified incubator containing 5% CO_2_. The cells were verified to be mycoplasma-free and passaged < 3 months. The inhibition of cell proliferation was measured by colorimetric 3-(4,5-dimethylthiazol-2-yl)-2,5-diphenyltetrazolium bromide (MTT) assay [45]. GI_50_ concentrations were determined using the CompuSyn software version 1.0 (ComboSyn, Inc., Paramus, NJ USA) (free download via www.combosyn.com, accessed on 30 May 2022), and statistical analyses were performed using the GraphPad PRISM software (version 6.01 for Windows, GraphPad Software, San Diego, California USA GraphPad Prism) using unpaired *t*-test. Data were considered to be statistically significant if *p* < 0.05 (*), *p* < 0.01 (**), *p* < 0.001(***) and *p* < 0.0001 (****).

## 4. Conclusions

With the lead compound curcumin, all the investigated ligands were characterized by a β-keto–enol group, suitable to bind hard Lewis acids such as Ga^3+^. Since one of the main drawbacks of curcumin is its high instability in physiological conditions, tightly related to its poor bioavailability, we synthesized seven new derivatives with improved stability in physiological environment (PBS pH 7.4, 37 °C). Estimating the stability under cell culture conditions would be remarkable to also predict potential therapeutic applications of the gallium complexes. In previous studies, the stability of gallium complexes with the lead compound curcumin was investigated in plasma and cellular cultures, demonstrating an increased stability of the metal complexes over the free ligand in these media and their ability to enter into the cells in vitro, as supported by confocal microscopy and ICP-MS analysis [46,47]. As it concerns the stability in a slightly acidic environment (pH 5–6) such as inside tumor cells [48], all gallium complexes are formed in acidic conditions (pH 4–6) and demonstrated to be stable in acid media, as suggested by the species distribution curves. However, it is not possible to exclude transchelation reactions; indeed, Ga^3+^ could be bound to the two metal-binding domains of transferrin with binding constants almost comparable to those of Fe^3+^ (log K_1_ = 20.3 (Ga^3+^), 22.8 (Fe^3+^) and log K_2_ = 19.3 (Ga^3+^), 21.7 (Fe^3+^)) at the normal plasma bicarbonate concentrations [21].

Additional issues in predicting the chemical stability and behavior in vivo are transmetalation reactions with endogenous metal ions. The high affinity of oxygen for strong Lewis acids such as Ga^3+^ reduces the incidence of transmetalation with bivalent metal ions such as Ca^2+^, Mg^2+^, Zn^2+^, while the exchange with Fe^3+^ cannot be excluded and should be estimated. Interestingly, iron is an essential nutrient the demand wherefore highly increases in cancer cells; consequently, limiting its availability can reduce cell proliferation, so iron chelators seem to have a high potential in cancer treatment [49,50]. The affinity for iron(III) of compounds **4** and **5** was comparable to that for gallium(III). Although we may not exclude transmetalation reactions with Fe(III), Ga^3+^ complexes of compounds **4** and **5** appear to be stable, given that iron homeostasis is finely regulated and the labile iron pool is negligible [51].

The main chemical features of the investigated ligands and their Ga^3+^ complexes are outlined in Table 4. The outcomes suggest that compounds **5** and, to a minor extent, **4** stand out as the most promising ones for potential therapeutic applications. In fact, the free ligand is more stable than curcumin, and a further increased stability is gained when Ga^3+^ complexes are formed. Compounds **4** and **5** are the strongest Ga^3+^-chelating agents, with pGa values similar to those of clinically established chelators (i.e., DFOA). In addition, the prevailing metal complex species are positively charged and remain unchanged with the switch in pH from physiological conditions (pH 7.4) to pathological ones, such as in tumor cells (pH 5–6), possibly favoring cellular uptake. Furthermore, upon the DPPH assay, compounds **4** and **5** and their Ga^3+^ complexes showed interesting antioxidant properties, with the IC_50_ values close to those of curcumin. As for similar compounds, the presence of a methoxy group in the ortho position to the phenolic one reduces the radical scavenging ability increasing the IC_50_ value [14]. The radical scavenging ability remains unaffected in the formation of Ga^3+^ complexes since the phenolic group remains untouched by metal coordination as previously observed for other derivatives [52].

According to the chemical characterization of these newly synthesized Ga(III) derivatives, molecules **3**, **4** and **5** were selected for MTT assays. The preliminary results pointed out that the conjugation of phthalimide significantly increases the antiproliferative activity in HCT116 cells, and among all, compound **5** showed major efficacy in terms of growth inhibitory activity (GI_50_) compared to curcumin. Further investigation would allow better characterizing this molecule as a potential therapeutic anticancer drug and could ascertain if its Ga(III) derivative can inhibit cancer-related cellular processes, such as clonal growth and migration, similarly to what we recently reported for compound **4 [26]**. It is also conceivable that gallium complexes might exert antimicrobial effects, as suggested by Seoung Choi et al. [53].

Finally, we demonstrated that compounds **6** and **7** can bind Ga^3+^ through the keto–enol moiety while the carboxylic group is not involved due to its spatial distance from the metal ion. The chemical structure of these compounds affects the overall stability constants of gallium complexes, reducing the Ga^3+^-sequestering ability. Nonetheless, this apparent flaw could be turned into an additional therapeutic property since the free carboxylate moiety could be exploited as a targeting vector for monocarboxylate transporters (MCTs), especially MCT1 that is overexpressed in several cancer cells, for instance, breast, lung and ovarian cancer cells [54,55]. 

## Figures and Tables

**Table 1:** Amount (molar percentage, %) of the keto–enol (KE) and diketo (KK) forms estimated based on the ^1^H NMR data acquired in MeOD-*d*_4_ at 298 K as reported in Experimental Section 3.5. * Percentage of KE includes the KE form of the Z isomer (6%).

Compound	KE (%)	KK (%)
**1**	96	4
**2**	88 *	12
**3**	45	55
**4**	47	53
**5**	70	30
**6**	25	75
**7**	25	75

**Table 2:** Overall protonation constants calculated from spectrophotometric data at 298 K using HypSpec software [34]; pK_a_ values were calculated from logβ values as follows: (HL) pK_a1_ = logβ_011_; (H_2_L) pK_a1_ = logβ_012_ − logβ_011_, pK_a2_ = logβ_011_; (H_3_L) pK_a1_ = logβ_013_ − logβ_12_; pK_a2_ = logβ_012_ − logβ_011_, pK_a3_ = logβ_011_; (H_4_L) pK_a1_ = logβ_14_ − logβ_13_, pK_a2_ = logβ_13_ − logβ_12_, pK_a3_ = logβ_12_ − logβ_12_, pK_a4_ = logβ_11_. * Refers to the keto–enol moiety dissociation.

**logβ_MLH_**	***1***	***2***	***3***	***4***	***5***	***6***	***7***
**logβ_011_**	10.212 (2)	10.903 (8)	10.88 (2)	10.78 (4)	10.44 (3)	11.42 (2)	12.22 (2)
**logβ_012_**	18.509 (3)		20.29 (2)	20.44 (6)	20.45 (2)	21.12 (6)	21.61 (5)
**logβ_013_**			28.84 (4)	29.44 (6)	29.58 (4)	29.76 (7)	29.98 (4)
**logβ_014_**						34.62 (5)	34.92 (9)
**pK_a1_**	8.297 (5)	10.903 (8) *	8.55 (4)	9.00 (10)	9.13 (6)	4.86 (12)	4.92 (13)
**pK_a2_**	10.212 (2) *		9.41 (4) *	9.66 (12) *	10.01 (5)	8.64 (13)	8.37 (9)
**pK_a3_**			10.88 (2)	10.78 (4)	10.44 (3)	9.70 (8) *	9.39 (7) *
**pK_a4_**						11.42 (2)	12.22 (2)

**Table 3:** Overall stability constants (β) and apparent formation constants (K) calculated from spectrophotometric data at 298 K using HypSpec software [36]. LogK values were estimated starting from the logβ_MLH_ ones according to the different number of acid groups as follows: (HL) logK_11_ = logβ_110_, logK_12_ = logβ_120_, logK_13_ = logβ_130_; (H_2_L) logK_11_ = logβ_111_ − logβ_11_, logK_12_ = logβ_122_ − 2logβ_11_, logK_13_ = logβ_133_ − 3logβ_11_; (H_3_L) logK_11_ = logβ_112_ − logβ_12_, logK_12_ = logβ_124_ − 2logβ_12_; (H_4_L) logK_11_ = logβ_112_ − logβ_12_; *pGa* (−log[Ga^3+^]_free_) was calculated at specific conditions ([Ga^3+^]_total_ = 1 μM, [L]_total_ = 10 μM, pH 7.4 at 25 °C).

	**1**	**2**	**3**	**4**	**5**	**6**	**7**
**H_x_L**	*H_2_L*	*HL*	*H_3_L*	*H_3_L*	*H_3_L*	*H_4_L*	*H_4_L*
**log** **β** **_110_**	11.78 (4)	8.129 (9)					
**log** **β** **_111_**	17.59 (3)						
**log** **β** **_112_**			28.28 (3)	28.48 (5)	28.54 (3)	27.79 (5)	28.29 (5)
**log** **β** **_113_**						34.09 (5)	36.51 (6)
**log** **β** **_120_**	20.30 (5)	15.185 (4)					
**log** **β** **_122_**	34.77(3)						
**log** **β** **_124_**			54.63 (5)	55.33 (5)	57.00 (1)		
**log** **β** **_130_**	28.37 (7)	20.45 (3)					
**log** **β** **_133_**	49.7 (4)						
**logK_11_**	7.38 (3)	8.129 (9)	7.99	8.04	8.09	6.67	6.68
**logK_12_**	14.35 (3)	15.185	14.05	14.45	16.1		
**logK_13_**	19.1 (4)	20.45					
**pGa**	19.0	13.1	20.8	21.5	23.2	14.0	15.3

**Table 4:** Summary of chemical features. * Percentage of the residual ligand after 4 h (ranking). ** Possible formation of gallium hydroxide species.

Ligand	Prevailing Tautomer (MeOD-*d*_4_)	Stability *	pGa	Prevailing Species (pH 5.5)	Prevailing Species (pH 7.4)
**1**	KE	77% (3)	19.0	[Ga(HL)_3_]	[Ga(L)_3_]^3−^
**2**	KE	83% (1)	13.1	[Ga(L)_3_]	[Ga(L)_3_]
**3**	KK	47% (6)	20.8	[Ga(H_2_L)_2_]^+^	[Ga(H_2_L)_2_]^+^
**4**	KK	42% (7)	21.5	[Ga(H_2_L)_2_]^+^	[Ga(H_2_L)_2_]^+^
**5**	KE	52% (5)	23.4	[Ga(H_2_L)_2_]^+^	[Ga(H_2_L)_2_]^+^
**6**	KK	73% (4)	14.0	[Ga(H_2_L)_2_]^+^	[Ga(H_2_L)_2_]^+^ **
**7**	KK	79% (2)	15.3	[Ga(H_2_L)_2_]^+^	[Ga(H_2_L)_2_]^+^ **

## Data Availability

Data is contained within the article and supplementary material.

## Supplementary Materials

The following supporting information can be downloaded at: https://www.mdpi.com/article/10.3390/ph15070854/s1, Figure S1: UV–Vis spectra of the investigated compounds at 25 °C in an aqueous solution (L = 50 μM). Figure S2: ^1^H NMR spectra of compound **7** in MeOD-*d*_4_ at 298 K (at 600 MHz) at the increasing addition of Ga^3+^. Figure S3: pH-metric spectrophotometric titration of the Ga^3+^:1 1:1 system in an aqueous solution (L = 50 μM; NaNO_3_ = 0.1 M, 298 K). Figure S4: Radical scavenging ability of derivatives **4** and **5** together with their Ga^3+^ metal complexes. Table S1: Liquid chromatography/mass spectrometry (LC/MS) results (*m/z*) for gallium(III) complexes.
