# Supplementary material for: Curcumin-Based β-Diketo Ligands for Ga3+: Thermodynamic Investigation of Potential Metal-Based Drugs
Source: Pharmaceuticals (Basel). 2022 Jul 12;15(7):854. doi: 10.3390/ph15070854 (PMC9321647; doi:10.3390/ph15070854)
Supplement: Supplementary file 1 [file pharmaceuticals-15-00854-s001.zip › pharmaceuticals-1771767-supplementary.pdf]

Article

Curcumin-based β-diketo ligands for Ga^{3+}: on the way to develop new antineoplastic agents

Debora Carrozza[1], Matteo Mari[1], Gianluca Malavasi[1], Ettore Venturi[1], Giulia Avino[2], Pier Cesare Capponi[3], Michele Iori[3], Sara Rubagotti[3], Silvia Belluti[4], Mattia Asti[3], Erika Ferrari[1,*]

1 Department of Chemical and Geological Sciences, University of Modena and Reggio Emilia, via G. Campi 103, 41125, Modena, Italy; erika.ferrari@unimore.it
2 Department of Chemical and Pharmaceutical Sciences, University of Trieste, via L. Giorgieri 1, 34127, Trieste, Italy.; gavino@units.it
3 Radiopharmaceutical Chemistry Section, Nuclear Medicine Unit, Azienda USL-IRCCS Reggio Emilia, via Amendola 2, 42122, Reggio Emilia, Italy.; mattia.asti@ausl.re.it
4 Department of Life Sciences, University of Modena and Reggio Emilia, via G. Campi 183, 41125, Modena, Italy; silvia.belluti@unimore.it

* Correspondence: erika.ferrari@unimore.it, Tel.: +39 059 205 8631

Figure S1.

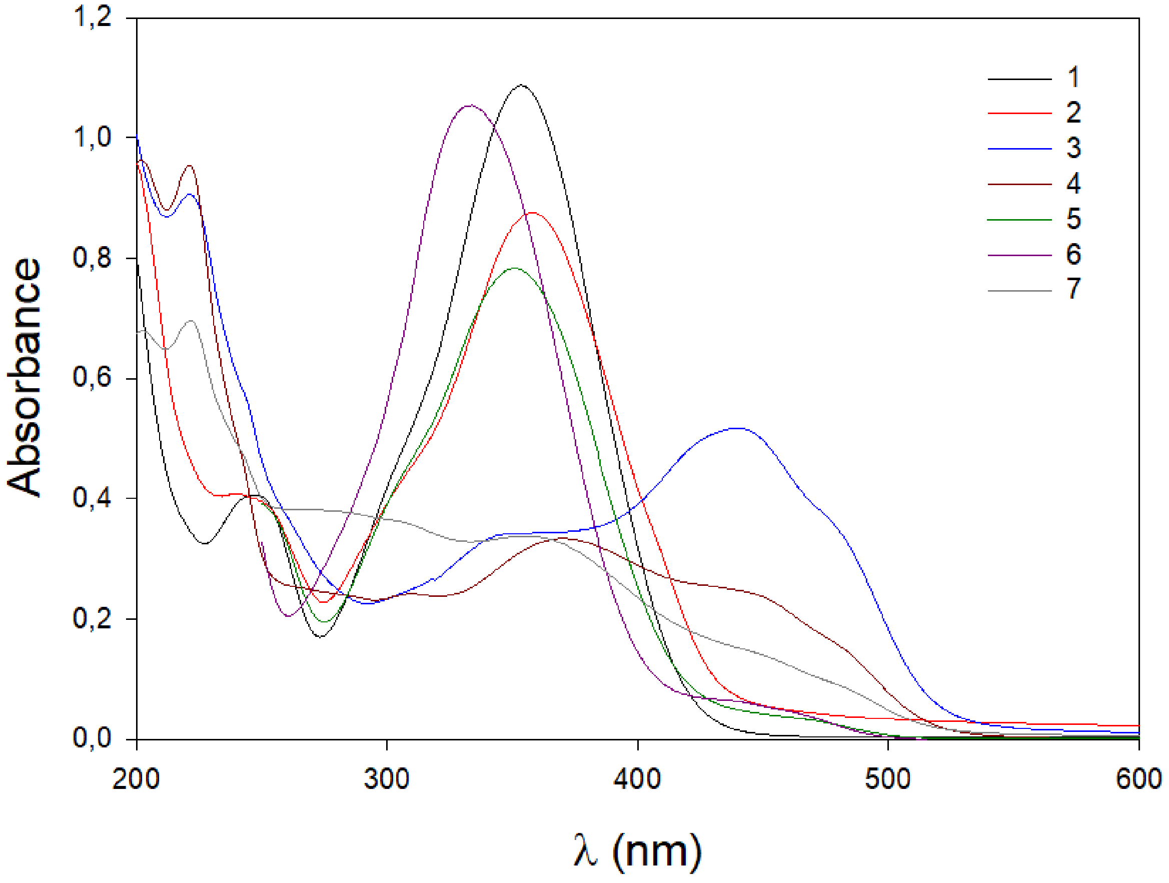

Figure S1. UV-Vis spectra of the investigated compounds at 25°C in aqueous solution ([L] = 50 μM, [$NaNO_3$] = 0.1 M, 298 K).

Figure S2.

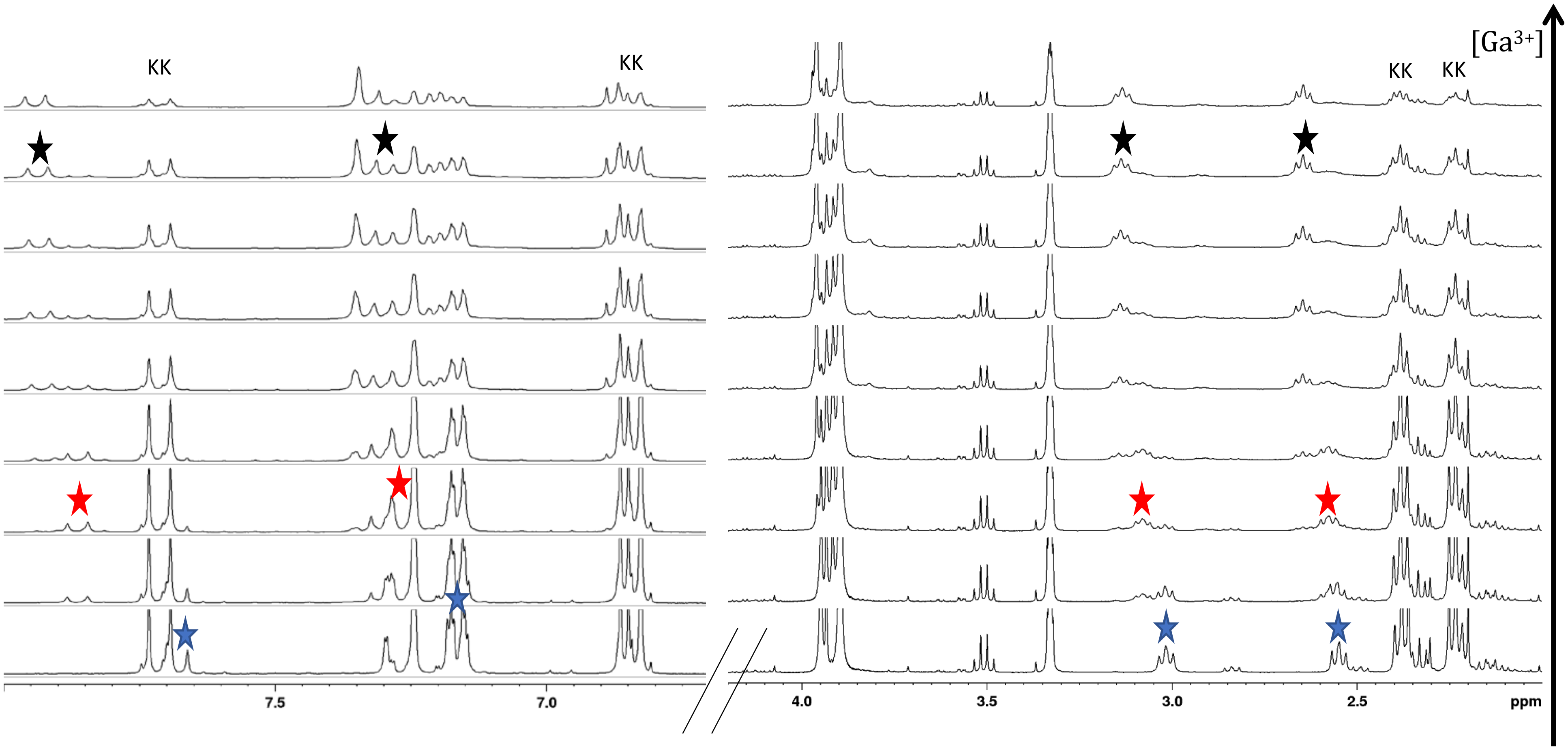

Figure S2. ^{1}H NMR spectra of compound **7** in MeOD-*d₄* at 298K (@600 MHz) at increasing addition of Ga^{3+} MeOD-*d₄* solution up to the metal-to-ligand 1:1.5 molar ratio. Stars highlights resonances of protons H-3, H-4, H-11 and H-12 in the free ligand (blue), M:L 1:2 complex (red) and M:L 1:1 complex (black). Analogous resonances for the di-keto form are labelled with KK and are unaffected by metal addition.

Figure S3.

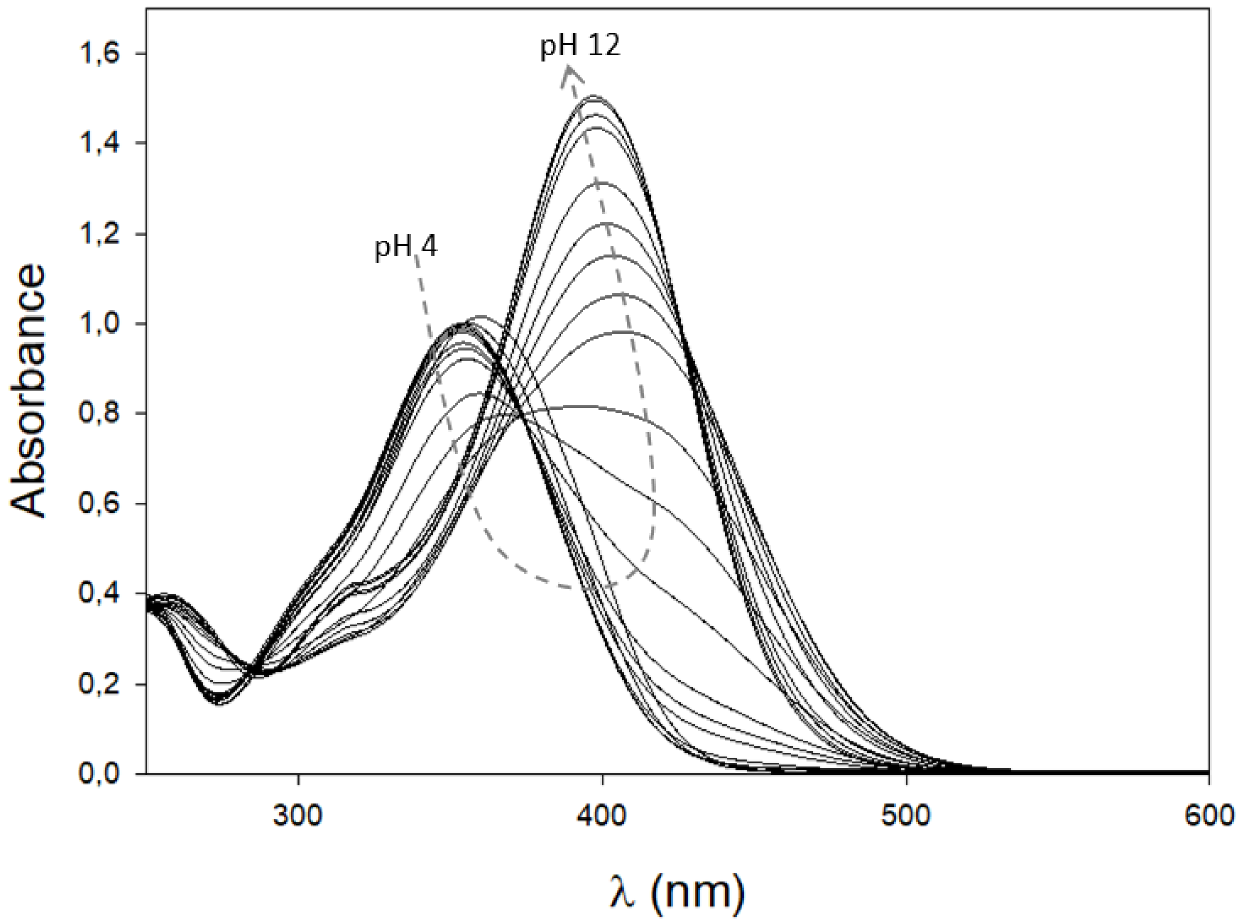

Figure S3. pH-metric spectrophotometric titration of Ga^{3+}:1 1:1 system in aqueous solution ([L] = 50μM; [$NaNO_3$] = 0.1 M, 298 K).

Figure S4.

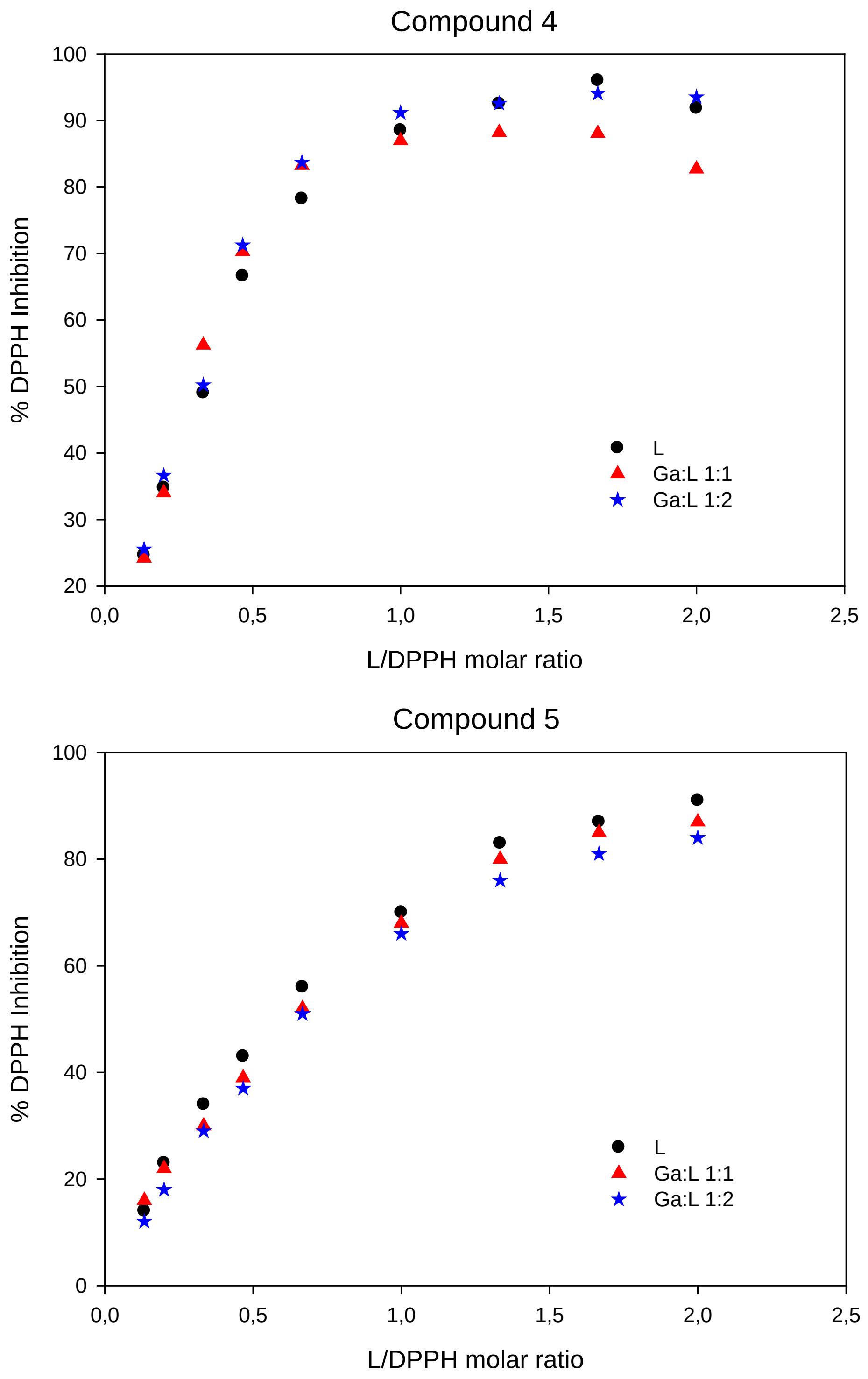

Figure S4. Radical scavenging ability of derivatives **4** and **5** together with their Ga^{3+} metal complexes. Percentage of inhibition of free DPPH radical ([DPPH$^{\cdot}$] = 60 μM) in the presence of the compound (free ligand (L) and gallium complexes) at increasing concentration. Compound concentration is expressed as molar ratio (L/DPPH).

Table S1. Liquid chromatography/mass spectrometry (LC/MS) results (*m*/*z*) for Gallium(III) complexes.

Compound	Formula	$[M+H]^+$
**1**	$[Ga(C_{13}H_{13}O_4)_2]^+$	536.11
**2**	$[Ga(C_{16}H_{18}BrO_4)_2]^+$	776.02
**3**	$[Ga(C_{29}H_{22}NO_6)_2]^+$	1046.30
**4**	$[Ga(C_{32}H_{28}NO_8)_2]^+$	1178.35
**5**	$[Ga(C_{30}H_{24}NO_6)_2]^+$	1058.29
**6**	$[GaC_{23}H_{22}O_6]^+$	448.09
**7**	$[GaC_{25}H_{26}O_8]^+$	524.12
